# Polycyclic Aromatic Hydrocarbons and Microbial Contamination in Traditional Pork Meat Products: Implications for Food Safety

**DOI:** 10.3390/microorganisms13122805

**Published:** 2025-12-09

**Authors:** Alexandra Tabaran, Oana Lucia Crisan-Reget, Dana Alina Magdas, Mihai Borzan, Sergiu Condor, Caroline Lǎcǎtuş, Sorin Daniel Dan

**Affiliations:** 1 Department of Animal Production and Food Safety, Faculty of Veterinary Medicine, University of Agriculture Sciences and Veterinary Medicine of Cluj-Napoca, 400684 Cluj-Napoca, Romania; oana.reget@usamvcluj.ro (O.L.C.-R.); mihai.borzan@usamvcluj.ro (M.B.); sergiu.condor@usamvcluj.ro (S.C.); sorindan@usamvcluj.ro (S.D.D.); 2 National Institute for Research and Development of Isotopic and Molecular Technologies—INCDTIM, 400293 Cluj-Napoca, Romania; alina.magdas@itim-cj.ro

**Keywords:** polycyclic aromatic hydrocarbons (PAHs), traditional, meat products, microbiological safety, benzo[a]pyrene

## Abstract

Traditional pork meat products produced through artisanal smoking and drying techniques are highly appreciated for their distinctive sensory characteristics; however, such practices may raise concerns regarding both chemical and microbiological safety. The present study aimed to assess the occurrence of selected polycyclic aromatic hydrocarbons (PAHs) and hygiene- and safety-related microorganisms in traditionally processed pork meat products collected from local markets and small-scale producers. A total of 140 samples were analyzed for four marker PAHs—benzo[a]pyrene (BaP), benz[a]anthracene (BaA), benzo[b]fluoranthene (BbF), and chrysene (Chr)—using gas chromatography–mass spectrometry (GC–MS). Microbiological contamination was evaluated through standard plate count techniques, and the presence of *Listeria monocytogenes* and *Salmonella* serovars was determined using selective isolation methods, followed by PCR confirmation of pathogenic strains. PAH concentrations varied widely: BaP (0.3–1.8 µg/kg), BaA (0.5–2.4 µg/kg), BbF (0.8–3.1 µg/kg) and Chr (0.4–2.0 µg/kg), with ΣPAH_4_ (Sum of PAH4, referring to the total concentration of the four-priority polycyclic aromatic hydrocarbons) ranging from 2.5 to 8.3 µg/kg. Smoked sausages showed the highest contamination (BaP: 1.8 µg/kg; ΣPAH_4_: 8.3 µg/kg), significantly exceeding levels in dry-cured ham (BaP: 1.2 µg/kg; ΣPAH_4_: 6.1 µg/kg) and smoked bacon (BaP: 0.9 µg/kg; ΣPAH_4_: 5.4 µg/kg) (Kruskal–Wallis, *p* < 0.0001). Although all samples complied with the EU ΣPAH_4_ limit (12 µg/kg), 15% exceeded the BaP limit of 2.0 µg/kg, primarily among artisanal sausages. Microbiological analyses revealed total coliform counts between 1.5 × 10^2^ and 6.2 × 10^4^ CFU/g, while Enterobacteriaceae ranged from 2.0 × 10^2^ to 4.9 × 10^4^ CFU/g. Samples obtained from unregulated producers exhibited higher bacterial loads, indicating suboptimal hygiene during processing and storage. A moderate positive correlation was identified between total coliform and Enterobacteriaceae counts (r = 0.59, *p* < 0.05). Moreover, *Salmonella* serovars was detected in ten sausage samples, and *Listeria monocytogenes* was confirmed in three samples of traditional products. Overall, the findings suggest that although PAH contamination generally complied with EU safety limits, occasional exceedances of benzo[a]pyrene and elevated microbial indicators underscore the need for stricter control of smoking parameters, fuel sources, and hygienic handling. Implementation of standardized smoking protocols and good manufacturing practices (GMP) is recommended to enhance the safety and quality of traditional pork meat products

## 1. Introduction

Romanian traditional pork meat products represent an essential component of the national gastronomic heritage, deeply rooted in rural culture and seasonal practices [1]. Products such as smoked bacon (slănină), dry-cured ham (jambon), and smoked sausages (cârnați) are prepared using long-established recipes, natural ingredients, and artisanal smoking methods [2,3,4]. Their characteristic flavor and texture are highly appreciated by consumers, who associate them with authenticity and cultural identity [1,2].

In rural and semi-rural areas, many households and small-scale producers continue to manufacture smoked pork products using traditional techniques. Smoking is typically carried out in rustic smokehouses using hardwoods such as beech or oak [5]. While these systems impart desirable sensory qualities, they lack the precise technological control of industrial smoking chambers, where smoke composition, temperature, humidity, and exposure time are regulated to reduce harmful contaminants such as polycyclic aromatic hydrocarbons (PAHs) [6,7]. In contrast, artisanal smoking depends heavily on producer experience and environmental conditions. Variations in combustion, oxygen flow, or smoking duration can lead to inconsistent smoke density and higher deposition of PAHs, while temperature fluctuations and uneven exposure may also influence microbial safety [8,9].

PAHs, particularly benzo[a]pyrene (BaP) and the sum of four marker compounds (ΣPAH_4_: BaP, benz[a]anthracene, benzo[b]fluoranthene, and chrysene), are among the most concerning chemical hazards in smoked foods because of their mutagenic and carcinogenic properties [10,11,12]. Exceedances of regulatory limits have been reported in European traditional meat products, including Romanian samples produced under uncontrolled smoking conditions or when inappropriate fuel sources are used [8,9].

Microbiological hazards likewise pose important risks in traditional meat processing. Hygiene indicator bacteria, such as total coliforms and *Enterobacteriaceae,* are frequently used to assess sanitary quality and the effectiveness of hygiene practices [13,14]. Elevated *Enterobacteriaceae* counts may indicate poor hygiene, post-processing contamination, or insufficient heat treatment, while total coliforms reflect issues related to cleaning, water quality, or handling [13]. Beyond these indicators, the presence of specific pathogens—particularly *Salmonella* serovars and *Listeria monocytogenes*—constitutes a direct food safety concern [14,15]. *Salmonella* remains a leading cause of foodborne outbreaks associated with pork, and *L. monocytogenes* presents a significant hazard in ready-to-eat (RTE) smoked meats due to its ability to survive refrigeration and persist in processing environments [16,17].

Smoked pork products remain highly valued by Romanian consumers for their traditional character and extended shelf life, yet the artisanal processing methods used in small-scale production may increase both chemical and microbiological risks. Traditional markets, where such products are commonly sold, often operate under limited sanitary supervision, and routine testing is less frequent than in industrial supply chains [2,9]. Consequently, contamination events may occur more often in these settings.

Previous studies on Romanian smoked meats have shown variable PAH levels and inconsistent microbiological quality. Tăbăran et al. (2018) [9] reported that although most samples complied with the European limit for BaP, several exceeded the ΣPAH_4_ threshold, reflecting variability in traditional smoking practices. Other surveys have found elevated coliform and Enterobacteriaceae counts in certain traditional sausages and smoked pork fats, and occasional detections of *Salmonella* and *L. monocytogenes* underline the need for improved hygiene during post-processing and retail stages [2,9].

This study provides a comprehensive assessment of both chemical (ΣPAH_4_) and microbiological (total coliforms, *Enterobacteriaceae*, *Salmonella*, and *Listeria monocytogenes*) safety parameters in a large set of traditionally processed Romanian pork products, including smoked sausages, dry-cured ham, and smoked bacon. Unlike previous regional surveys, our work stratifies PAH contamination by product type, allowing a more detailed risk evaluation. Additionally, the study explores correlations between product characteristics and contamination levels, offering new insights into the factors influencing both chemical and microbiological hazards in traditional pork processing.

## 2. Materials and Methods

### 2.1. Sample Collection

A total of 140 traditionally processed pork meat products were collected for analysis. The sample set consisted of smoked sausages (*n* = 75), dry-cured ham (*n* = 46), and smoked bacon (*n* = 19). The products were obtained from local markets (registered producers) and small-scale artisanal producers (family-owned, locally sold, direct-to-consumer sales, in rural/household settings that use traditional or artisanal methods, sometimes without standardized procedures). This small-scale local producers often lack official oversight, such as hygiene inspections, quality control, safety certification, or production standards. These producers were located across geographically diverse rural areas of Transylvania, Romania, specifically within Cluj, Alba, Sălaj, and Mureș counties. Collection sites were selected to represent typical regional production practices and to capture variability in raw materials, processing techniques, and storage conditions.

#### Sampling Procedure

Sampling was conducted over a one-year period (November 2024–November 2025) to ensure consistency in product handling and environmental conditions, and to include the Christmas season, when these products experience increased demand. At each collection point, we have selected products directly from the producer’s display or cold storage area.

Each meat product was aseptically removed from its original storage environment, avoiding direct hand contact. The products selected were freshly produced products, not stored in polyethylene (PE) or other packaging materials. Immediately after removal, representative portions (approximately 500 g) were transferred into sterile, food-grade polyethylene sampling bags. Bags were sealed using sterile closures to prevent leakage and to maintain microbiological integrity during transport.

All sealed samples were placed inside insulated portable coolers containing refrigerant ice packs to maintain a constant low temperature. Throughout transport, the temperature was monitored using portable thermometers to ensure it remained at 4 ± 1 °C, preventing bacterial growth or biochemical changes that might alter analytical results.

Samples were transported directly from the collection sites to the laboratory. The duration between sampling and laboratory arrival was minimized (typically within 2–4 h, depending on the location site), and coolers were not opened during transit except when necessary for temperature checks.

Upon arrival at the laboratory, sample integrity was verified. Each bag was inspected for proper sealing, evidence of leakage, or signs of temperature abuse. Two samples were excluded from the research because of possible contamination during transport and were not considered in the statistics.

Samples were then logged into the laboratory information system, assigned identification codes, and stored in a refrigerated environment at 4 ± 1 °C until analysis. To ensure consistency and prevent degradation, all analyses were conducted within 24 h of sample collection.

For each product type, a representative portion (35 g) from the central section of each piece was used. Product surfaces were trimmed, if necessary, using sterile scalpels or knives to eliminate external contamination that could interfere with microbiological or physicochemical assessments. For ham, a slice was taken from the mid-muscle region, avoiding the peripheral ends to reduce variability caused by drying or smoke penetration gradients. For smoked bacon, the sample consisted of both the meat layer and the adipose layer, taken from the middle section of the slab. The thickness of the subcutaneous fat layer in the collected samples ranged between 5 and 10 mm, corresponding to the natural anatomical structure of the products. This layer was not trimmed, as it forms an integral part of the edible portion and is relevant for both heterocyclic aromatic compounds/polycyclic aromatic hydrocarbons and microbiological evaluation. For sausage product, a central cross-section was collected to ensure representation of the internal matrix. The diameter of the sausages was 2.5 cm, measured at the midpoint of each sausage. All sausages were filled exclusively in natural membranes (natural pork casings). No collagen or cellulose casings were used for any of the products included in the analysis. The natural membrane was removed prior to analysis for both PAH determination and microbiological assessment, in accordance with our general protocol to analyze only the edible fraction. Each sample was cut into small pieces including both lean and fat tissue.

### 2.2. Determination of Polycyclic Aromatic Hydrocarbons (PAHs)

#### 2.2.1. Sample Preparation

Approximately 10 g of homogenized sample was weighed and subjected to solvent extraction using n-hexane and acetone (1:1, *v*/*v*). The extract was cleaned by solid-phase extraction (SPE) on silica cartridges (Supelclean LC-Si, Supelco, Bellefonte, PA, USA), evaporated under nitrogen to near dryness, and reconstituted in 1 mL of n-hexane prior to analysis.

PAH Extraction and Purification:

Solid phase extraction (SPE) purification was performed using a standardized protocol. Silica gel columns were pre-conditioned with hexane, and the sample extracts were loaded onto the columns. PAHs were then eluted using a hexane/dichloromethane mixture (3:1, *v*/*v*), optimized for maximal recovery of the four marker compounds (BaP, BaA, BbF, and Chr). Extraction efficiency was verified by spiking blank meat samples with known PAH concentrations, yielding average recoveries of 85–95%, consistent with published methods and EU performance criteria.

The eluate was evaporated under nitrogen to near dryness and reconstituted in 1 mL of n-hexane prior to analysis.

#### 2.2.2. Instrumental Analysis (GC–MS)

Quantification of the four marker PAHs—benzo[a]pyrene (BaP), benz[a]anthracene (BaA), benzo[b]fluoranthene (BbF), and chrysene (Chr)—was performed using a gas chromatograph coupled with a mass spectrometer (PerkinElmer Clarus 500 GC–MS, PerkinElmer Inc., Shelton, CT, USA). Separation was achieved on a DB-5MS capillary column (30 m × 0.25 mm × 0.25 µm film thickness). The injector temperature was set at 280 °C, and helium was used as the carrier gas at a constant flow rate of 1.0 mL/min. The oven temperature program was as follows: 60 °C (held 2 min), increased to 280 °C at 10 °C/min, and held for 10 min. The MS was operated in selected ion monitoring (SIM) mode. Calibration was performed using certified PAH standards (Supelco, Bellefonte, PA, USA), and results were expressed as µg/kg fresh weight.

### 2.3. Microbiological Analysis

#### 2.3.1. Enumeration of Indicator Microorganisms

Each sample (10 g) was homogenized in 90 mL of sterile Buffered Peptone Water (BPW) using a stomacher (Stomacher 400, Seward, Worthing, West Sussex, UK) for 2 min. Serial dilutions were plated on Plate Count Agar (PCA) for total aerobic mesophilic counts and incubated at 30 °C for 72 h.

Total coliforms were enumerated on Violet Red Bile Agar (VRBA, Sigma Aldrich, München, Germany) at 37 °C for 24 h, while Enterobacteriaceae were determined on Violet Red Bile Glucose Agar (VRBGA) under the same conditions. Results were expressed as colony-forming units per gram (CFU/g).

#### 2.3.2. Detection of Pathogenic Bacteria

*Salmonella* serovars

The detection of Salmonella spp. was performed following the procedures outlined in ISO 6579-1:2017. Briefly, 25 g of each sample were homogenized in 225 mL of Buffered Peptone Water (BPW) and subjected to non-selective pre-enrichment at 37 °C for approximately 18 h to facilitate the recovery of sublethally injured cells. Subsequently, an aliquot of the pre-enrichment culture was transferred to Rappaport–Vassiliadis (RV) broth (Oxoid, France) for selective enrichment, which supports the preferential proliferation of Salmonella under inhibitory conditions.

Following selective enrichment, cultures were streaked onto Xylose Lysine Deoxycholate (XLD) agar (Oxoid, Dardilly, France) for isolation. Presumptive Salmonella colonies—typically characterized by red colonies with black centers—were subjected to biochemical confirmation. Biochemical verification was performed using Triple Sugar Iron (TSI) agar and Lysine Iron Agar (LIA) (Thermo Fisher Scientific, Waltham, MA, USA), enabling assessment of sugar fermentation profiles, lysine decarboxylation, and hydrogen sulfide production. Colonies exhibiting characteristic Salmonella reactions were considered confirmed isolates.

*Listeria monocytogenes*

The detection of *Listeria monocytogenes* followed ISO 11290-1:2017. A 25-g portion of each sample was aseptically transferred into Half-Fraser broth (Oxoid, Basingstoke, Hampshire UK) for primary enrichment and incubated at 30 °C for 24 h to promote resuscitation of stressed Listeria cells. A measured volume of the primary enrichment was subsequently inoculated into Fraser broth for secondary enrichment at 37 °C for an additional 24 h. The selective components of Fraser broth provide enhanced suppression of competing microorganisms, while allowing the proliferation of *L. monocytogenes*.

Secondary enrichment cultures were streaked onto Oxford agar (Oxoid, Basingstoke, Hampshire, UK) for selective isolation. Presumptive colonies, typically displaying grey-green coloration with black halos resulting from esculin hydrolysis, were further examined through confirmatory biochemical assays. Confirmation included the catalase test, verifying catalase activity characteristic of Listeria species, and the CAMP test, which assesses the hemolytic synergism typical of *L. monocytogenes*. Colonies exhibiting the expected biochemical reactions were considered confirmed (catalase, CAMP test).

### 2.4. Molecular Confirmation by PCR

Presumptive *Salmonella* and *Listeria monocytogenes* isolates were confirmed by polymerase chain reaction (PCR). DNA was extracted using a previously published method by Mihaiu et al., 2014 [18]. Specific gene targets were amplified using standard primers: *inv*A for *Salmonella* serovars [19] and *hly*A for *Listeria monocytogenes* [20] (Table 1). Amplification products were visualized on 1.5% agarose gels stained with ethidium bromide under UV illumination.

### 2.5. Statistical Analysis

The sample sizes of the three product categories—smoked sausages (*n* = 75), dry-cured ham (*n* = 46), and smoked bacon (*n* = 19)—reflect the availability and market share of traditional pork products in the studied region during the sampling period. Smoked sausages are more commonly produced and sold by both registered and small-scale artisanal producers, whereas smoked bacon is less frequently available, particularly from rural small-scale producers. To account for this imbalance, non-parametric statistical methods were applied for group comparisons, and confidence intervals for proportions and means were calculated using approaches robust to small sample sizes, such as Wilson intervals. While the smaller number of smoked bacon samples limits the precision of estimates for this category, the analyses remain representative of the products available in local markets, and the observed trends are consistent with regional production characteristics and previous studies.

Descriptive statistics (mean, standard deviation, and range) were calculated for all measured parameters. Pearson correlation coefficients were determined to assess associations between microbial indicators. Inter-group differences in PAH concentrations among the three product categories were assessed using the non-parametric Kruskal–Wallis test, followed by post hoc pairwise comparisons where appropriate. Effect sizes were calculated to quantify the magnitude of differences, and significance was set at *p* < 0.05. Data were processed using IBM SPSS Statistics v.26.0 (IBM Corp., Armonk, NY, USA), with significance set at *p* < 0.05.

## 3. Results

### 3.1. Polycyclic Aromatic Hydrocarbons (PAHs) in Traditional Pork Products

The analysis of 140 traditionally processed pork meat products revealed variable concentrations of the four marker PAHs—benzo[a]pyrene (BaP), benz[a]anthracene (BaA), benzo[b]fluoranthene (BbF), and chrysene (Chr). The mean concentrations of individual compounds ranged from 0.3 to 1.8 µg/kg for BaP, 0.5 to 2.4 µg/kg for BaA, 0.8 to 3.1 µg/kg for BbF, and 0.4 to 2.0 µg/kg for Chr. The total ΣPAH_4_ concentrations varied between 2.5 and 8.3 µg/kg across samples.

Approximately 15% of the analyzed products exceeded the European Union (EU) maximum permissible limit for BaP (2.0 µg/kg), while all samples complied with the regulatory limit of 12.0 µg/kg for ΣPAH_4_ in smoked meat products (Regulation (EU) No 835/2011) [21]. The highest BaP levels were detected in dry-cured smoked sausages, particularly those obtained from small-scale artisanal producers, suggesting variability in smoking temperature and fuel source.

**Figure 1:**
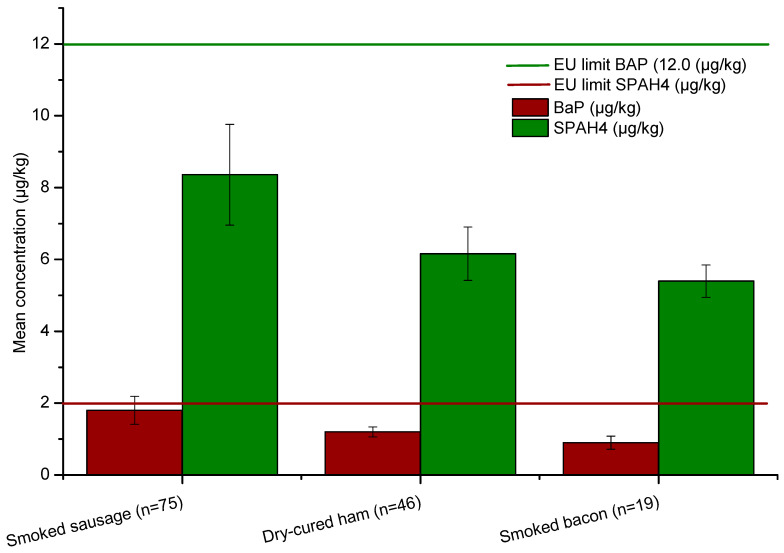
Mean concentration of BaP and ΣPAH_4_ within the three categories of samples studied.

The comparative analysis of PAH contamination levels (Figure 1) revealed clear differences among the three categories of traditionally processed pork products. Smoked sausages (*n* = 75) exhibited the highest mean concentrations for both benzo[a]pyrene (BaP) and total ΣPAH_4_, with average values of 1.8 µg/kg and 8.3 µg/kg, respectively. These concentrations, although below the European Union (EU) regulatory limits (2.0 µg/kg for BaP and 12.0 µg/kg for ΣPAH_4_), were notably higher than those observed in dry-cured ham (BaP: 1.2 µg/kg; ΣPAH_4_: 6.1 µg/kg) and smoked bacon (BaP: 0.9 µg/kg; ΣPAH_4_: 5.4 µg/kg).

Formal statistical analysis using the Kruskal–Wallis test confirmed significant differences in PAH contamination among the three categories of traditionally processed pork products. For benzo[a]pyrene (BaP), the test indicated a highly significant difference (H = 48.21, *p* < 0.0001), with smoked sausages exhibiting the highest concentrations, followed by dry-cured ham and smoked bacon. Similarly, total ΣPAH_4_ concentrations differed significantly across product types (H = 88.60, *p* < 0.0001), reinforcing that smoked sausages consistently contained higher levels of marker PAHs.

Stratified analysis of BaP exceedances showed that smoked sausages registered the highest proportion of samples above the EU BaP limit (6/75; 8.0%, 95% CI: 3.7–16.4%), followed by dry-cured ham (3/46; 6.5%, 95% CI: 2.2–17.5%) and smoked bacon (1/19; 5.3%, 95% CI: 0.9–24.6%). Although the overall exceedance rate was 15% across all products, this product-specific breakdown clarifies that sausages present the highest relative risk of BaP non-compliance.

The elevated PAH levels in smoked sausages (Table 2) are likely attributable to the longer smoking duration and higher exposure to combustion residues, typical of artisanal sausage production. In contrast, dry-cured ham and smoked bacon undergo milder or shorter smoking phases, which may limit PAH deposition. Despite the variability among product types, none of the mean values exceeded EU safety limits, confirming that overall contamination remained within acceptable regulatory thresholds. However, the relatively high BaP concentration in smoked sausages—approaching the maximum permissible level—suggests that inconsistent smoking parameters, such as temperature control and fuel type, may contribute to sporadic exceedances in individual samples.

### 3.2. Microbiological Quality and Safety Indicators

Microbiological examination revealed a wide range of bacterial contamination levels among samples. The total coliform counts ranged from 1.5 × 10^2^ to 6.2 × 10^4^ CFU/g, while Enterobacteriaceae counts varied between 2.0 × 10^2^ and 4.9 × 10^4^ CFU/g. Samples obtained from unregulated vendors and open markets generally exhibited higher microbial loads compared to those originating from licensed producers. A moderate positive correlation (r = 0.59, *p* < 0.05) was observed between total coliform and *Enterobacteriaceae* counts, suggesting similar contamination sources and inadequate hygiene during processing or storage.

Regarding pathogen detection, ten samples (7.1%) of traditionally processed sausages tested positive for *Salmonella* se, while three samples (2.1%) were positive for *Listeria monocytogenes*. All positive *Listeria* samples were collected from the same category of smoked sausages obtained from small-scale artisanal producers lacking standardized hygiene control systems. PCR confirmation of pathogenic strains was successful for all presumptive isolates.

No significant correlation (*p* > 0.05) was found between PAH concentrations and microbial contamination levels, suggesting that the smoking process, while contributing to PAH formation, did not significantly influence the survival of hygiene indicator bacteria. However, samples exhibiting exceedances in BaP concentrations often coincided with higher total viable counts, implying that inconsistent smoking conditions may simultaneously favor both chemical contamination and microbial persistence.

## 4. Discussion

The present study evaluated both chemical (polycyclic aromatic hydrocarbons, PAHs) and microbiological safety indicators in 140 traditionally processed pork products across three categories (smoked sausages, dry-cured ham, smoked bacon). The dual assessment provides insight into simultaneous chemical and microbial hazards associated with artisan smoking/curing practices.

In this study, only the four EU marker PAHs—benzo[a]pyrene (BaP), benz[a]anthracene (BaA), benzo[b]fluoranthene (BbF), and chrysene (Chr)—were quantified, as they constitute the ΣPAH_4_ regulatory indicator used for smoked meat products in the European Union [21]. Although a broader panel of 16 EPA-PAHs could provide a more comprehensive profile of contamination, the ΣPAH_4_ subset captures the most toxicologically relevant compounds and allows direct comparison with EU regulatory limits and previous studies. A limitation of this approach is that potential contributions from other PAHs outside the ΣPAH_4_ panel are not included in the risk assessment, which may slightly underestimate total PAH exposure. Nevertheless, the selected marker PAHs remain the most widely accepted indicators for food safety evaluation in smoked meats.

The concentration ranges obtained for the marker PAHs suggest that traditional smoking practices generate measurable but generally moderate levels of contamination across the sampled products. The distribution of mean values—highest BaP and ΣPAH_4_ in smoked sausages, followed by dry-cured ham and smoked bacon—indicates that certain technological steps specific to sausage processing may facilitate greater PAH deposition. Although around 15% of samples exceeded the EU limit for BaP, none surpassed the regulatory threshold for ΣPAH_4_ in smoked meats [21], implying that exceedances tend to be compound-specific rather than indicative of an overall high PAH burden.

When placed in a broader European context, these findings align with the literature showing that artisanal smoking methods typically result in higher PAH contamination compared with industrially standardized systems. Polish data, for example, demonstrate that sausages smoked for more than 60 min and exposed to direct heat accumulated significantly higher PAH levels than their industrial counterparts [6]. Similarly, another investigation reported median concentrations of 9.73 µg/kg for traditionally smoked loin and 52.71 µg/kg for traditionally smoked bacon, whereas industrially smoked products from the same study remained below 1 µg/kg [22]. In this light, the PAH values observed in our samples fall within the lower–middle range of those reported for traditional smoking, yet they reflect the same underlying pattern of elevated contamination under less-controlled conditions.

Previous reviews have emphasized that PAH formation in smoked meats is strongly influenced by processing parameters, including fuel type, smoke exposure duration, temperature, and oxygen availability [23]. The comparatively higher PAH levels detected in smoked sausages may therefore reflect a combination of longer or more intense smoke exposure, greater contact with combustion residues, or fluctuations in smoking chamber conditions often characteristic of small-scale artisanal operations.

From a risk-assessment perspective, the PAH concentrations in our study should be considered in the context of existing evaluations. Prior assessments of smoked meats, such as those conducted in China, have shown that even when PAH levels are relatively high, the resulting lifetime cancer risks and hazard indices frequently remain below established regulatory thresholds (HI < 1; CR < 1 × 10^−5^) [24]. While such comparisons do not negate the need to reduce PAH exposure, they suggest that the contamination levels observed here may not pose substantial health risks for consumers under typical dietary patterns.

The proximity of some samples in our study to the BaP regulatory limit (~1.8 µg/kg mean, some >2.0 µg/kg) indicates that individual high-contamination products may present elevated risk, particularly when consumption is frequent.

While our study provides valuable insights into this topic, several limitations should be acknowledged. Key factors such as specific smoking parameters, the type of wood used, and the structural characteristics of the smoking room were not systematically recorded. Similarly, intrinsic product properties, including water activity (aw) and pH, were not measured. These omissions were largely due to the large number of small-scale producers involved and the inherent variability and typicity of traditional processing methods, which made standardized measurements challenging. As a result, our ability to fully explain the causal mechanisms underlying the observed outcomes is limited. Future studies should aim to address these factors, which would strengthen causal interpretations and enhance understanding of traditional smoking practices.

Microbiological analysis revealed total coliform counts from 1.5 × 10^2^ to 6.2 × 10^4^ CFU/g, and *Enterobacteriaceae* from 2.0 × 10^2^ to 4.9 × 10^4^ CFU/g. A moderate positive correlation (r = 0.59, *p* < 0.05) between the two indicators suggests shared contamination sources, likely processing surface contamination, cross-contamination or storage hygiene lapses. Pathogenic bacteria were detected: *Salmonella* serovars in 7.1% of samples (10/140) and *Listeria monocytogenes* in 2.1% (3/140); all Listeria-positive samples derived from smoked sausages produced by small-scale artisanal producers lacking standard hygiene controls. Most of previous research emphasize that traditional meat processing, especially by small-scale operators, is associated with higher microbial contamination and pathogen risk when compared to industrialized systems [25,26,27,28]. For example, fermentation and smoking processes can mitigate some microbial hazards, but only when combined with good hygiene practices and validated process controls [29].

The detection of pathogens in our samples underscores this vulnerability: even though the chemical hazard (PAHs) may be under control on average, microbial hazards are still present in the same product category (smoked sausages), particularly when processing is less regulated.

## 5. Conclusions

This study assessed the chemical and microbiological safety of traditional Romanian pork products produced using artisanal smoking and curing methods. Although ΣPAH_4_ values remained within EU regulatory limits, a small proportion of samples exceeded the permissible level for benzo[a]pyrene, and occasional detections of *Salmonella* serovars and *Listeria monocytogenes* highlighted vulnerabilities in hygiene control. These findings indicate that traditional products can meet food safety requirements when adequate process management and good manufacturing practices are applied. Continued efforts to improve smoking control and strengthen producer training remain essential to ensuring both the safety and authenticity of traditional Romanian meat products.

## Figures and Tables

**Table 1:** Primers, PCR Conditions, and Controls for Detection of *Salmonella* serovars (*inv*A) and *Listeria monocytogenes* (*hly*A) in Traditional Pork Products.

Targeted Gene	Primer Sequence (5′–3′)	Amplicon Size (bp)	PCR Conditions	Positive Control
*inv*A (*Salmonella* serovars)	F:ACAGTGCTCGTTTACGACCTGA R:AGACGACTGGTACTGATCGATAAT	284	Initial denaturation: 95 °C 5 min 35 cycles: 95 °C 30 s, 58 °C 30 s, 72 °C 45 s Final extension: 72 °C 7 min	*S. enterica* ATCC 14028
*hly*A (*Listeria monocytogenes*)	F:GCAGTTGCAAGCGCTTGGAG R:GCAACGTATCCTCCAGAGTGATCG	456	Initial denaturation: 95 °C 5 min 35 cycles: 95 °C 30 s, 55 °C 30 s, 72 °C 45 s Final extension: 72 °C 7 min	*L. monocytogenes* ATCC 19115

**Table 2:** PAH Concentrations, Microbial Loads, and Pathogen Occurrence in Traditionally Processed Pork Products.

Product Type	BaP (±SD) (µg/kg)	ΣPAH_4_ (±SD) (µg/kg)	Mean Total Coliforms (CFU/g)	Mean Enterobacteriaceae (CFU/g)	Pathogens Detected
Smoked Sausages	1.8 ± 0.39	8.36 ± 1.4	3.2 × 10^4^	2.8 × 10^4^	Salmonella (7.1%) Listeria (2.1%)
Dry-Cured Ham	1.2 ± 0.14	6.16 ± 0.74	1.5 × 10^4^	1.2 × 10^4^	None detected
Smoked Bacon	0.9 ± 0.18	5.4 ± 0.45	1.2 × 10^4^	1.0 × 10^4^	None detected

## Data Availability

The original contributions presented in this study are included in the article. Further inquiries can be directed to the corresponding author.
